# Evaluation of the Genmark ePlex® and QIAstat-Dx® respiratory pathogen panels in detecting bacterial targets in lower respiratory tract specimens

**DOI:** 10.1186/s12866-021-02289-w

**Published:** 2021-08-26

**Authors:** Suzanne A. V. van Asten, Stefan A. Boers, Jolanda D. F. de Groot, R. Schuurman, Eric C. J. Claas

**Affiliations:** 1 Department of Medical Microbiology, Leiden University Medical Center, PO Box 9600, Leiden, 2300 RC The Netherlands; 2 Department of Medical Microbiology, University Medical Center Utrecht, Utrecht, The Netherlands

**Keywords:** ePlex, QIAstat-Dx, Molecular diagnostics, Respiratory tract infections, Syndromic testing

## Abstract

**Background:**

The ePlex® and QIAstat-Dx® respiratory pathogen panels detect multiple respiratory pathogens, mainly viruses but also *Legionella pneumophila*, *Mycoplasma pneumoniae* and *Bordetella pertussis*. The assays have been marketed for use in nasopharyngeal swab specimens. For diagnosing bacterial pneumonia, lower respiratory tract (LRT) specimens are indicated. Aim of this study was to evaluate the performance of these syndromic panels for these three bacterial targets in samples from the LRT. Fifty-six specimens were collected from our repositories, five negative samples and fifty-one samples which had been previously tested positive with the routine diagnostic real-time PCR assays for *Legionella* spp. (*N* = 20), *Bordetella* spp. (*N* = 16) or *M. pneumoniae* (*N* = 15).

**Results:**

The QIAstat-Dx Respiratory Panel V2 (RP) assay detected all of the *L. pneumophila* and *B. pertussis* positive samples but only 11/15 (73.3 %) of the *M. pneumoniae* targets. The ePlex Respiratory Pathogen Panel (RPP) assay detected 10/14 (71.4 %) of the *L. pneumophila* targets, 8/12 (66.7 %) of the *B. pertussis* positive samples and 13/15 (86.7 %) of the *M. pneumoniae* targets.

**Conclusions:**

No false-positive results were reported for all three bacterial pathogens by both assays. The clinical performance of both assays depended highly on the bacterial load in the sample and the type of specimen under investigation.

## Background

Community-acquired respiratory tract infections are a leading cause of hospitalization worldwide and a significant cause of mortality, especially in vulnerable patient groups. Some bacterial pathogens, like *Legionella pneumophila*, are critical to detect because they represent important epidemiologic challenges and can cause serious complications that require treatment strategies different from standard empiric regimens [1].

There is substantial progress in the development of syndromic testing platforms for respiratory infections, gastroenteritis and even neurological infections [2]. These assays are able to rapidly detect multiple pathogens associated to clinical syndromes, including viruses, bacteria and parasites [2–4]. The GenMark Respiratory Pathogen Panel (RPP) assay on the ePlex instrument was evaluated in several clinical studies [5, 6] and showed excellent overall agreement of over 95 % compared to laboratory-developed (multiplex) real-time PCR assays (LDTs) in samples with cycle threshold (C_T_) values < 35. In a recent clinical application study, the assay also resulted in improved prescription of antimicrobial therapy, a reduction in isolation days of admitted patients, and detection of pathogens that were not requested to investigate by the clinician [7]. Another rapid cartridge-based assay, the QIAstat-Dx® RP assay (Qiagen®) has become available for detection of 21 respiratory pathogens. The clinical performance of this assay was evaluated in a multicentre retrospective study [8] and showed good performance in comparison to the ePlex® RPP assay. On top of that, the QIAstat-Dx® RP assay also provides C_T_ values and thus a semi-quantitative indication of the pathogen load within the samples.

Both the ePlex® RPP and QIAstat-Dx® RP assays have the limitation that CE *in vitro* diagnostics (CE-IVD) and FDA clearance has only been provided for nasopharyngeal swab samples. Where the majority of viruses included in these panels cause upper respiratory tract infections (URTI), bacterial targets as *Legionella pneumophila* and *Mycoplasma pneumoniae* affect the lower respiratory tract (LRT) and therefore sputum or bronchoalveolar lavage fluid samples (BAL) seem more appropriate to diagnose infection [9]. Data on the performance of the ePlex® RPP and QIAstat-DX® RP assays for detecting *L. pneumophila*, *M. pneumoniae and B. pertussis* targets is limited. All these targets are reported in the European CE/IVD ePlex cartridges, but the Legionella and Bordetella targets are not reported in FDA cleared cartridges. These three targets were either absent or hardly evaluated in the available clinical studies [5, 6, 8]. Therefore, the value of these parameters in clinical practice, especially in off-label use on LRT specimens, remains largely unknown. The objective of the present study is to expand data on the clinical performance of the ePlex® RPP and QIAstat-Dx® RP assays to detect *L. pneumophila*, *B. pertussis* and *M. pneumoniae* in LRT specimens.

## Results

Of the 56 samples analyzed with the ePlex® RPP assay one result was invalid after repeat testing, a mucopurulent sputum sample originally containing a very low level of *L. pneumophila* DNA (C_T_ 38.5) that tested negative with the QIAstat-Dx® RP assay. In the QIAstat-Dx® analyzer all samples were evaluable; in four cases repeat testing was required to obtain a valid result. In one of the latter samples, *M. pneumoniae* was detected with a C_T_ value of 28.1 but the IC failed.

The performance characteristics of the included samples are presented in Table 1. In one of the nasopharyngeal swab samples only the *Bordetella* IS1001 target was detected by the LDT assay, indicating that this sample was positive for *B. parapertussis*. Three samples tested positive for only the *Bordetella* IS481 target (range 24.6–38.8) and were not detected with the ePlex® RPP assay, while the QIAstat-Dx® RP assay detected all three of them.

**Table 1:** Comparison of results of bacterial targets detection by the ePlex® RPP assay and the QIAstat-Dx® RP assay

Bacterial target	Median LDT C_T_ value (range)	Interpretation	Detected by ePlex® RPP assay	Detected by QIAstat-Dx® RP assay	Median QIAstat-Dx® RP assay C_T_ value (range)
*Bordetella* spp. IS481/IS1002 IS481 IS1001	23.8 (6.5–29.3) 35.4 (24.6–38.8) 30.6	*B. pertussis* positive *B. species* *B. parapertussis*	8/12 0/3 0/1	12/12 3/3 0/1	24.9 (15.5–35.1) 37.1 (27.4–37.0)
*Legionella* spp. *L. pneumophila*	27.5 (14.7–33.0) 30.1 (23.8–35.4)	*L.* non-*pneumophila* *L. pneumophila*	0/5 10/14^a^	0/5 14/14^a^	31.2 (25.9–35.5)
*M. pneumoniae*	26.4 (20.7–39.0)	*M. pneumoniae*	13/15	11/15	28.2 (22.1–35.6)

^a^One sample not evaluable despite repeated testing

The QIAstat-Dx® RP assay detected all of the *L. pneumophila* and *B. pertussis* targets (*n* = 14 and *n* = 12, respectively), but only 11/15 (73.3 %) of the *M. pneumoniae* targets. The four targets that were not detected by the QIAstat-Dx® RP assay had a C_T_ value > 32.5 as determined by LDT assays. The ePlex® RPP assay detected 10/14 (71.4 %) of the *L. pneumophila* targets, 8/12 (66.7 %) of the *B. pertussis* targets and 13/15 (86.7 %) of the *M. pneumoniae* targets. The range of the C_T_ values of the targets that were not detected by the ePlex® RPP assay was 25.6–36.6, five targets that were missed had C_T_ values < 30 (including four *B. pertussis* and one *L. pneumophila* target).

The negative control samples and the five *Legionella* non-*pneumophila* positive samples were reported negative for all pathogens by both ePlex® RPP and QIAstat-Dx® RP assays. Additional pathogens that were detected in these samples were rhinovirus/enterovirus (*N* = 7), coronavirus HKU1 (*N* = 2), adenovirus (*N* = 3), RSV (*N* = 2) and human metapneumovirus (*N* = 1).

## Discussion

This study demonstrates the application of two commercially available molecular-method-based syndromic panels for *off-label* detection of bacterial targets in LRT samples. Though the number of negative samples is small, we did not detect any false positivity of the assays (100 % specificity). The analytical sensitivity however, differed for the three bacterial targets tested and seemed to depend mainly on the bacterial load in the samples (based on LDT C_T_ values). This finding is in line with previous studies that evaluated the detection of viral pathogens in clinical LRT samples using multiplex assays [5, 8, 10, 11].

As *L. pneumophila* is an important pathogen for community acquired respiratory infections, with a specific treatment regimen, accuracy and speed of diagnosis is crucial. The ePlex® RPP assay did not detect four of the *L. pneumophila* positive LRT samples while the QIAstat-Dx® RP assay detected all of them. The *L. pneumophila* samples that were missed by the ePlex® RPP assay were all sputa. With a reported limit of detection of 30 CFU/ml (package insert), the viscosity of the material or the extraction method of the assay might have affected detection of the pathogen. Previous studies have shown that caution should be exercised when interpreting test results from the ePlex® RPP assay that are derived from sputum samples [5, 8]. Basically, a positive result is positive, but interpretation of a negative result may not rule out a Legionella infection.

Both assays demonstrate moderate/impaired sensitivity for the detection of *M. pneumoniae* in the evaluated clinical samples with lower bacterial loads (C_T_ > 30). Previous studies have shown high positive percentage agreement for detecting *M. pneumoniae* with the ePlex® RPP assay compared to the BioFire® FilmArray® (93.3 %) (6) and with the BioFire® FilmArray® compared to the SOC FilmArray RP (95.8 %) (2). This discrepancy might be caused by: (1) the use of different materials in this study (as the other studies only used nasopharyngeal swabs), (2) by lower bacterial loads in our samples, (3) by a difference in the extraction methods, or by (4) differences in the analytical sensitivities of the LDT method used in these comparisons. However, since clinical diagnosis of *M. pneumoniae* is often difficult and DNA positivity in infected patients has been reported to be very short [12], coincidental detection of this pathogen using syndromic panels might still be useful [13, 14].

The difference in the detection of *B. pertussis* between the QIAstat-Dx® RP assay and the ePlex® RPP assay is remarkable (100 vs. 66.7 % respectively). The target with the greatest analytical sensitivity to detect *B. pertussis* is the insertion sequence IS481 [15]. The utilization of a single-copy pertussis toxin promotor target (ptxP) in several multiplex panels has been shown to be less sensitive for the detection of *B. pertussis* compared to assays based on the multicopy IS481 insertion sequence [16]. The ePlex RPP panel uses a specific single gene (potentially ptxP) as target, while the QIAstat-Dx® RP assay uses the IS481 multicopy sequence, which is less specific as it can be present in other Bordetella species as well. In addition, the input volume of the QIAstat-DX® RP assay is 300 µl versus 200 µl for ePlex®. One other study evaluating the Filmarray for respiratory pathogens in children found complete correlation (100 %) with an LDT for *B. pertussis* for nine clinical samples, but these were all nasopharyngeal swabs with a high bacterial load [11].

## Conclusions

Altogether, it can be concluded that both ePlex® RPP and QIAstat-Dx® RP assay are able to provide reliable positive diagnostic results on LRT specimens although the decreased limit of detection in these samples may result in false negative results with low bacterial load (CT > 30). Because of the small number of samples and the retrospective nature of our results, a future, bigger designed trial needs to confirm these data.

## Methods

### Clinical samples

This study is a collaboration between two university medical centers in the Netherlands (Leiden University Medical Center, LUMC, Leiden and University Medical Center Utrecht, UMCU, Utrecht). We selected a total of 61 respiratory samples from our repositories collected between January 2007 and December 2019. These samples were collected from patients of all ages and both sexes, presenting with signs and/or symptoms of respiratory tract infections from which a positive results was obtained using the diagnostic real-time PCR assays, that were implemented under the ISO15189 international standard for medical laboratories [17, 18].

The selected samples included sputum samples (*N* = 26), bronchoalveolar lavage fluid samples (*N* = 11), bronchial secretion samples (*N* = 1), nasopharyngeal swabs collected with an E-Swab (Copan) containing 1 ml of liquid Amies media (*N* = 7), nasopharyngeal aspirates (*N* = 2) and throat swabs collected with an ESwab (Copan) or UTM (Copan) containing 2 ml of liquid Amies media (*N* = 14). As part of our routine diagnostic workflow, all sputum samples, bronchoalveolar lavage fluid samples, secretions and aspirates were 1:5 diluted in phosphate-buffered saline (PBS) and homogenized by bead-beating prior to testing because of their viscosity. No pre-treatment was performed on swab samples. All materials were anonymized after thawing and no clinical data was collected, therefore ethical approval for this study was waived.

All samples had been prospectively tested with LDTs for respiratory pathogens and aliquots stored at -80 °C were used for the current study. The selected samples consisted of 20 samples positive for *Legionella* spp. of which 15 for *L. pneumophila*, 16 samples positive for *Bordetella* spp., 15 samples for *M. pneumoniae* and 5 samples which had been demonstrated completely negative for each of these pathogens. Initially, our *Bordetella pertussis* PCR assay only targeted the IS481 sequence, that also can be detected in *B. holmesii* and *B. bronchiseptica.* Adding a second PCR targeting the IS1002 fragment increased the specificity for *B. pertussis* [19]. Samples were considered *B. pertussis* positive when both IS481 and IS1002 targets were detected. When samples were only positive for IS481 only, they were considered *Bordetella species* positive.

## ePlex® RPP and QIAstat-Dx® RP assays

The ePlex® RPP assay is based on a closed electrowetting technology by which droplets of sample and reagents can be moved efficiently within a network of electrodes in the cartridge. The eSensor technology is able to detect influenza A/B virus, parainfluenza virus (1–4), respiratory syncytial virus A/B, adenovirus, human coronavirus (229E/HKU1/NL83/OC43), Middle East respiratory syndrome coronavirus (MERS), human bocavirus, human metapneumovirus, human rhinovirus/enterovirus (combined), *Chlamydia pneumoniae, L. pneumophila, B. pertussis* and *M. pneumoniae.* Importantly, the *B. pertussis* assay in the ePlex targets a specific gene for *B. pertussis* and not the multicopy IS481. After pre-treatment, 200 µL of the respiratory sample was pipetted in a tube with buffer (supplied by the manufacturer) and, after vortexing, transferred into the ePlex® RPP cartridge and tested. After approximately 90 min the results of the different pathogens were reported as either positive, negative or invalid (e.g. internal control (IC) failure). If the test reported an invalid result or an error occurred, the samples were retested with a new cartridge.

The QIAstat-Dx® analyzer, combined with the QIAstat-Dx® RP assay cartridges, uses real-time multiplex PCRs to detect respiratory pathogens in a closed system. Real-time amplification signals are interpreted by the integrated software and reported in approximately 70 min. The respiratory pathogens detected include the same pathogens as the ePlex® RPP assay with the exception of *C. pneumoniae* and MERS. According to manufacturer’s instructions, 300 µL of the prepared respiratory sample was transferred into the QIAstat-Dx® RP assay cartridge and loaded into the analyzer. The results were reported with the corresponding C_T_ value. If an invalid result was reported (e.g. IC failure) or an error occurred with the cartridge, the samples were retested.

## Data Availability

The datasets used and/or analysed during the current study are available from the corresponding author on reasonable request.
